# Scurvy Extinct? Think Again!

**DOI:** 10.5005/jp-journals-10005-1017

**Published:** 2009-12-26

**Authors:** Vela D Desai, Shweta Hegde, Durgesh N Bailoor, Neelkant Patil

**Affiliations:** 1 Associate Professor, Department of Oral Medicine and Radiology, Jaipur Dental College, Jaipur, Rajasthan, India; 2 Postgraduate Student, Department of Oral Medicine and Radiology, Jaipur Dental College, Jaipur, Rajasthan, India; 3 Principal, Professor and Head, Department of Oral Medicine and Radiology, Jaipur Dental College, Jaipur, Rajasthan, India; 4 Assistant Professor, Department of Oral Medicine and Radiology, Jaipur Dental College, Jaipur, Rajasthan, India

**Keywords:** Diet, scurvy, vitamin C deficiency, gingival hemorrhage, gingival enlargement.

## Abstract

Scurvy is still seen sporadically in the developed world. Scurvy, a dietary disease due to the deficient intake of vitamin C, is uncommon in the pediatric population. Scurvy occurs as a result of decreased vitamin C consumption or absorption.

We present the case of a 6-year-old boy visiting our department with bleeding gums, musculoskeletal pain, and weakness. Four days after starting oral vitamin C supplementation, there was significant improvement in the patient’s gingival appearance and general health. The clinical presentation and laboratory investigation (Hemoglobin %, total blood picture) , together with the dramatic therapeutic response to ascorbic acid administration, confirmed the diagnosis of scurvy. Scurvy can be missed unless oral and general physicians maintain a high index of suspicion. Therefore it is time to wonder if scurvy is extinct yet.

## CASE REPORT

**Fig. 1.**
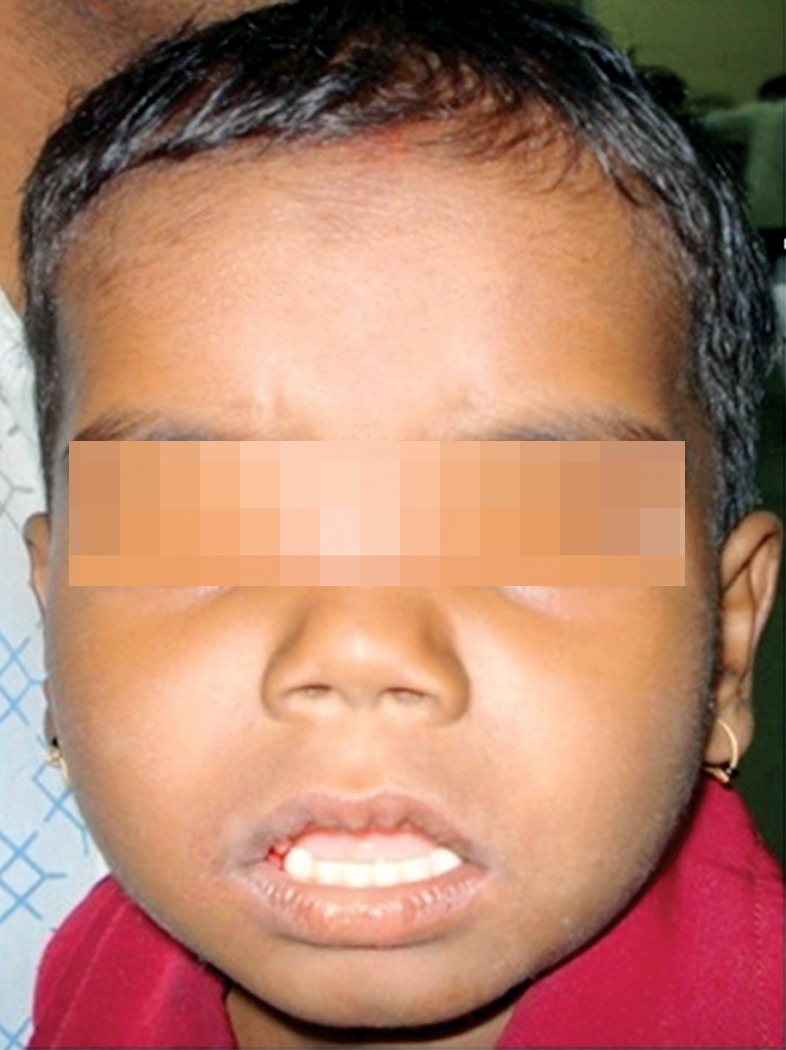
Six years old boy with normal extraoral profile

A 6-year-old boy reported to our department with a chief complaint of severe pain with progressive bleeding gums and musculoskeletal pain of 1 month duration (Fig. 1).

Patient was extremely irritable, apprehensive, uncooperative and had to be carried to the OPD. His mother revealed history of poor dietary habits devoid of any fruits or vegetables. Patient was not on any medications and there was no history of rash, fever, respiratory difficulty or symptoms of bulbar dysfunction. Patient has been unable to brush his teeth since 15 days due to pain and bleeding in the gums.

On examination, the patient looked moderately well, afebrile. His weight and height were on the 65th and 60th percentiles, respectively. He had mild weakness of hip flexion and extension bilaterally, but normal muscle tone and deep tendon reflexes. The results of rest of his neurological examination were normal. There was no hepatosplenomegaly or lymphadenopathy.

**Fig. 2A and B:**
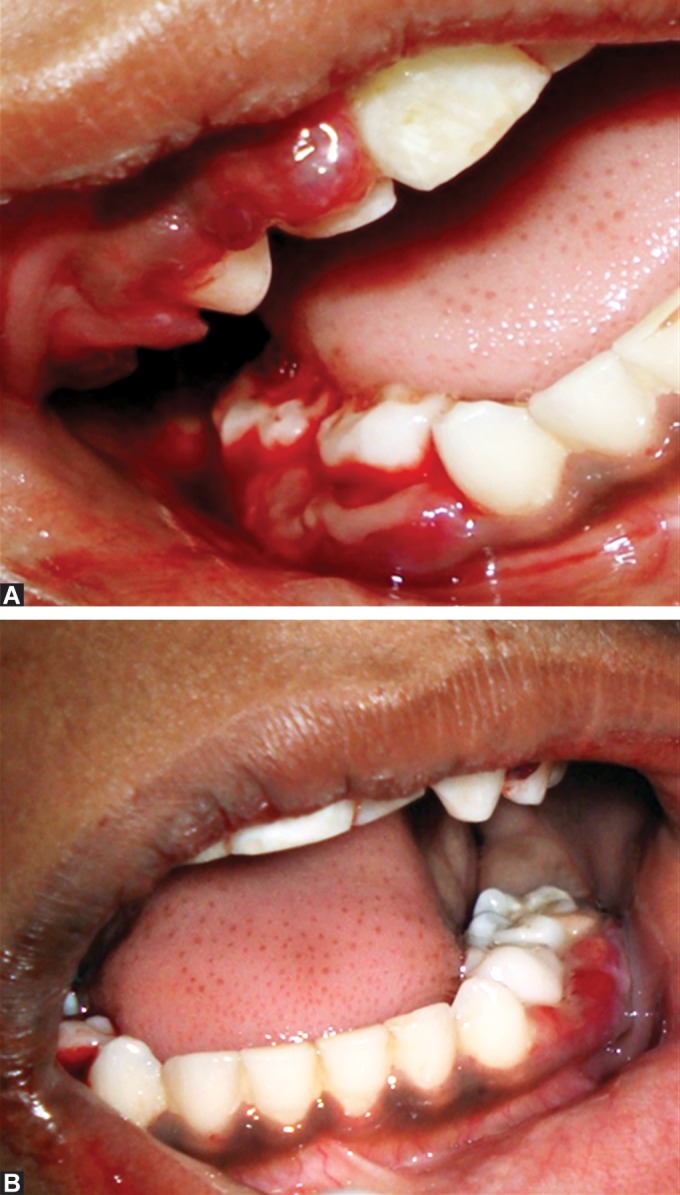
Swollen, hemorrhagic gingiva in relation to most of the teeth

**Fig. 3A and B:**
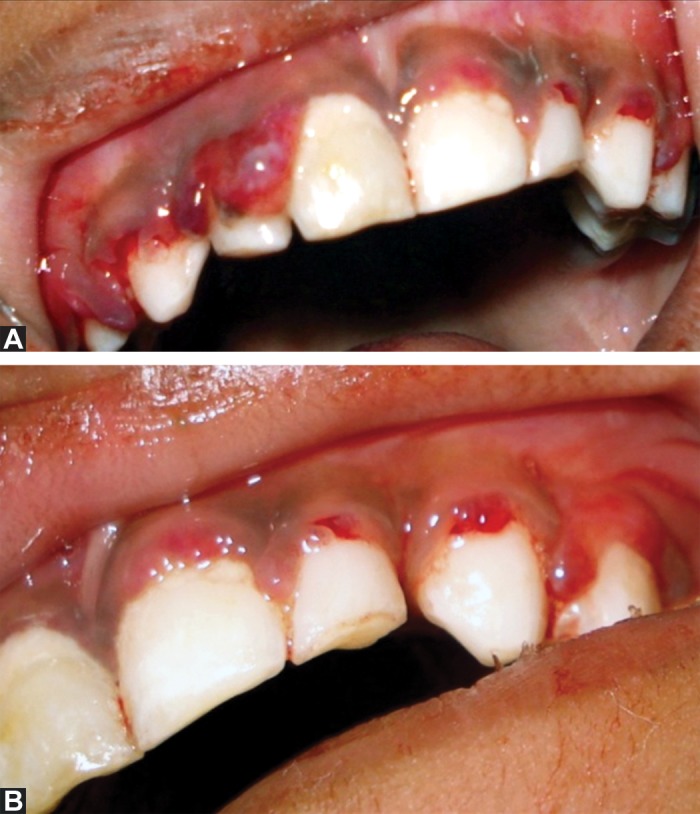
Enlarged gingival without any contributing local factors

Intraoral examination revealed hemorrhagic, swollen, spongy, tender gingival in relation to all teeth which bled spontaneously and also on slight provocation (Figs 2A and 2B). Dentition comprised mostly of deciduous teeth except for permanent maxillary anterior incisors and had no carious lesions in any teeth. Local factors such as plaque and calculus were minimal and not contributory to the gingival presentation (Figs 3A and 3B).

**Fig. 4.**
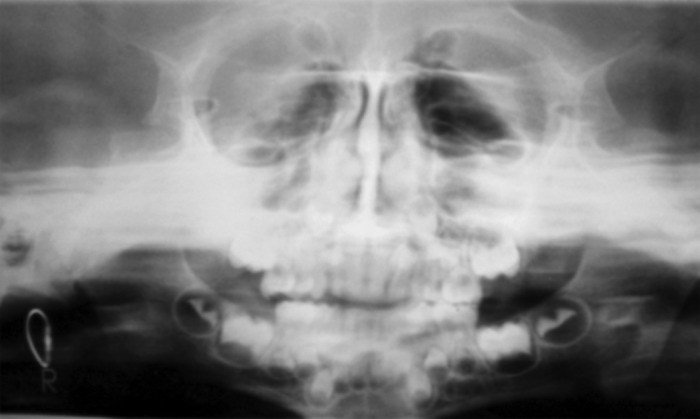
OPG revealed mixed dentition with no apparent changes in bone and no periapical pathology

Scurvy was made as a provisional diagnosis on the basis of dietary history and clinical presentation, although prepubertal gingival enlargement and leukemic enlargement were considered for differentiadiagnosis. The patient was further subjected to laboratory hematological investigations to rule out any other bleeding diathesis and radiological investigations to rule out periapical pathology (Fig. 4). Hematological report revealed low hemoglobin percentage of 9.3 mg% but peripheral smear was normal.

**Fig. 5.**
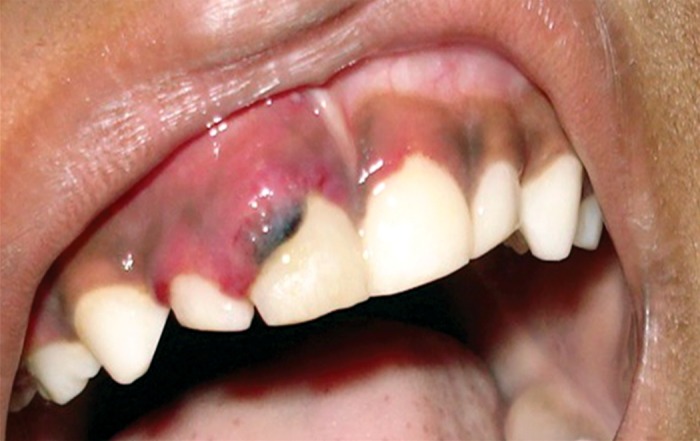
Marked reduction in gingival enlargement and hemorrhage within a period of 4 days

**Fig. 6.**
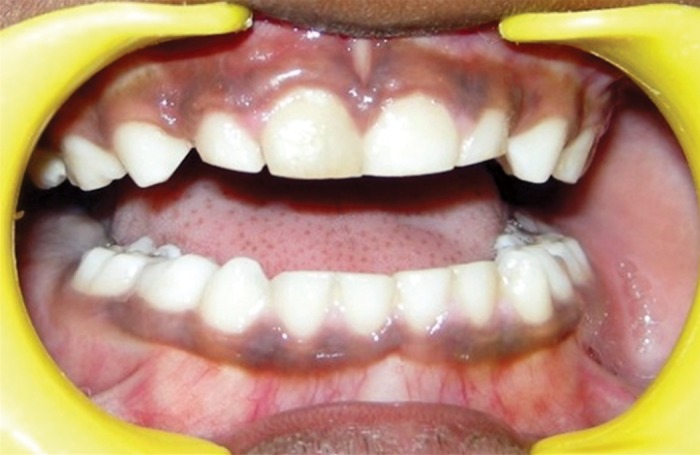
Complete resolution of inflammatory component and normal appearance of gingival after 15 days

Patient was administered chewable vitamin C tablets 500 mg OD, and a combination of vitamin C syrup with iron and folic acid, 2 ml TDS for 7 days and nutritional counseling (to the parent) was done to include vitamin C rich fruits and vegetables in his diet. Patient’s response to the therapy was dramatic. Patient was cooperative and calm on his next visit as he was relieved of the severe pain intraorally as well as of knee and calf muscles. There was an incredible change in the gingival health with marked reduction in the hemorrhagic appearance and inflammatory component within 4 days (Fig. 5). Patient was recalled every fortnight to monitor his gingival conditions and overall health (Fig. 6). 

## DISCUSSION

Although scurvy is rare in the developed world, it still occurs sporadically.

The word scurvy probably originated from the middle low German word schorbuk which came from schoren, "break", and buk, "belly" referring to the phenomenon observed among the seafarers during the long sea voyages of the 15th to 18th centuries, where old healed scars and wounds would disintegrate, some leading to a "ruptured belly".^1^-^3^ Scurvy is the nutritional deficiency state associated with lack of ascorbic acid levels which leads to suppression of collagen synthesis and the synthesis of defective collagen among other metabolic derangements.^4^ Ascorbic acid is also required for many other biological processes such as synthesis of carnitine and neurotransmitters (norepinephrine), gastrointestinal absorption of iron, prostaglandin metabolism and cellular immunity.^3^

The recommended dietary allowance (RDA)5 for adults is 60 mg/day of vitamin C. This will maintain a total body pool of about 1500 mg, preventing scurvy. A minimum daily dose of 10 mg is sufficient to avoid scurvy. Adults should receive 100 mg 3-5 times a day up to 4 grams followed by 100 mg/day for a week, and infants and children, 10-25 mg 3 times a day.

Vitamin C is readily available from citrus fruits, green vegetables (e.g. broccoli, brussel sprouts), potatoes and tomatoes. Some meats, such as kidney and liver, are also good sources.^6^-^8^

Diagnosis of scurvy is a clinical one. It is based on specific clinical features, supported by a consistent diet history and the rapid resolution following vitamin C supplementation. In our case report, the patient presented with some of the signs and symptoms typical of vitamin C deficiency (Table 1). Patients are treated based on clinical manifestations. Symptoms usually disappear within 3-5 days, and most physical findings resolve in 1-2 weeks as was observed in our case. Scurvy is usually not isolated, and other nutritional deficiencies should therefore be sought in newly diagnosed cases. A high index of suspicion remains the mainstay for diagnosing scurvy in order to avoid expensive and lengthy laboratory work-up, including aggressive procedures. The laboratory investigations for less typical cases include ascorbic acid concentration,^8^ serum ascorbic acid level below 11 mg/dl,^9^ leukocyte ascorbate level^8^ and ascorbic acid tolerance test.^10^

The prognosis of scurvy is excellent, and the response to vitamin C is often good. Table 2 describes the systemic complications in extreme cases which may have to be ruled out if patient does not respond to therapeutic vitamin C.

Late manifestations of scurvy are dyspnea, peripheral edema, hemarthroses, sicca syndrome, femoral neuropathy due to hemorrhage into the femoral sheath and hemopericardium.^3^ The mechanism for the cardiorespiratory events is unclear and is postulated to arise from impaired vasoconstriction to adrenergic stimuli leading to syncope, refractory hypotension, and death. Groups at risk (Table 3) are those with poor or unbalanced diets.

Dentists and physicians should be aware of the clinical presentations of vitamin C deficiency, because the presentation of the patient with scurvy may be subtle. Recognizing the disease requires heightened vigilance; however, when patients with scurvy are diagnosed early, the condition can be readily treated.

**Table:** TABLE 1: Depicting the function of vitamin C and their result in defective or deficient production

*Vitamin C functions*		*Defective or deficient production causes*
Hydroxylation of collagen		Blood vessel fragility, poor wound healing^6^
		*Oral cavity:* Hemorrhagic gingiva, loss of teeth, xerostomia, halitosis
		*Skin:* Petechiae to perifollicular ecchymosis, palpable purpura due to edema, bleeding, dry skin, hyperkeratotic papules (thighs, legs, buttocks)
Biosynthesis of carnitine which is a metabolic source of energy at skeletal and myocardial muscles^9^		Muscle weakness of lower extremities, fatigue, myalgia,^8^ pseudoparalysis
*Promotes iron absorption:* Reduces iron into ferrous form and aids in GI absorption		*Anemia^10^:* Normocytic, normochromic, iron and folic acid deficiency; GI and soft tissue bleeding, hemolysis, nails develop into splinter hemorrhage

**Table:** TABLE 2: Severe clinical manifestations of vitamin C deficiency^3^

*Systemic manifestations*		*Clinical presentation*
Rheumatologic		Painful hemarthrosis, subperiosteal hemorrhage, Barlow syndrome in infants (pain and immobilized posture with hip and bone in semiflexion).
Cardiac		Cardiac enlargement due to high output anemia resulting in congestive cardiac failure, hemopericardium
Ophthalmic manifestation		Conjunctival hemorrhage and fundus changes, and cotton wool spots, papilledema, optic nerve atrophy, Sjogren like syndrome

**Table:** TABLE 3: Risk groups^3^ in scurvy

Nutritional deficiency states for example		Food faddists, allergy to multiple fruits and vegetable products, poverty
Oxidative states such as in		Diabetes, smoking, myocardial infarction
Gastrointestinal disorders for exmaple		Colitis, malabsorption
Cancer patients on chemotherapy11 like		Interleukin II, interferon
Patients on hemodialysis in		End stage Renal disease
Psychiatric disorders		Depression, schizophrenia, anorexia
Immune compromised states		Acquired immunodeficiency syndrome

This case report suggests that in any child presenting with musculoskeletal symptoms, the possibility of a nutritional cause, particularly vitamin C deficiency, secondary to abnormal eating patterns be considered before undertaking extensive investigations. All health care professionals must make a proactive effort such as inclusion of dietary counselling as a part of routine treatment plan to eradicate scurvy.
